# Addressing Disparities for Intersectional BIPOC Communities: The Hood Medicine Initiative Case Study

**DOI:** 10.1016/j.eclinm.2021.101199

**Published:** 2021-11-15

**Authors:** Emily Hotez, Shanice Hudson, Anchuen Cho, Charles Senteio, Jonathan White, Doug Slaughter, Peter Hotez, Quinn Chipley

**Affiliations:** a David Geffen School of Medicine, University of California, Los Angeles (UCLA), 911 Broxton Ave, Los Angeles, CA, 90024; b Hood Medicine Initiative, Inc, P.O. Box 55458, Indianapolis, IN 46205; c Rutgers University School of Communication and Information, 4 Huntington Street, New Brunswick, NJ 08901; d National School of Tropical Medicine, Baylor College of Medicine (BCM), One Baylor Plaza, Houston, Texas 77030

The COVID-19 pandemic shed light on and exacerbated health disparities experienced by Black, Indigenous, and People of Color (BIPOC) [1], [2], [3], particularly BIPOC with multiple and intersectional marginalized identities (BIPOC/MIMI) [4]. Indeed, disparate access to vaccination during the early rollout and persistent disparate access to quality health care are especially pronounced for BIPOC with intellectual and/or developmental disabilities, those who identify as lesbian, gay, bisexual, transgender, and/or queer, as well as myriad other marginalized groups [5]. During the pandemic, Hood Medicine Initiative (HMI)—a nonprofit public health collective comprised of experts in science, policy, technology, and marketing—was born out of the need to tackle health disparities for BIPOC/MIMI. In response to *The Lancet's* commitment to anti-racism, we highlight HMI as a scalable model for combating the heightened inequities for BIPOC/MIMI spurred by the pandemic.

In June 2020, the racial inequities amplified by the pandemic converged with the murder of George Floyd and the expansion of the Black Lives Matter movement. Despite heightened public discourse on their increased risk of COVID-19 infection, hospitalization, and mortality [1], public health officials were challenged to effectively communicate to BIPOC/MIMI. Indeed, traditional public health messaging has historically lacked the nuance required to reach a marginalized populace, attributable to both systematic medical malfeasance and a lack of data-driven communication approaches. It was therefore not surprising that tracking by the Kaiser Family Foundation and other researchers confirmed significant rates of vaccine hesitancy and inequitable vaccine access in BIPOC communities [1], [2], [3].

Given the unmet needs of BIPOC/MIMI, HMI coalesced around a mission to develop innovative ways of using media and technology to improve communication of the benefits of vaccination. HMI began to create and disseminate live podcasts (https://tinyurl.com/Hood-Med-Chats-YouTube) featuring health professionals and community activists to target these groups directly. HMI quickly expanded its programming over the course of the pandemic to include infographics and compelling public health messaging.

Currently, HMI develops culturally tailored public health messaging for BIPOC/MIMI. HMI forges authentic collaborations with grassroots stakeholders to influence public health behaviors and aid diverse communities in practical and actionable ways. These efforts are complemented by efforts to create, translate, and disseminate sustainable research-driven solutions for diverse BIPOC sub-segments. HMI seeks to integrate these efforts across health disciplines—including maternal and child health, preventive medicine, genetic counseling, and community education—to address widespread disparities in BIPOC/MIMI.

To further disseminate this tailored approach, HMI developed the HMI Framework (Figure 1)—geared towards scientists, practitioners, and policymakers—that describes the process of developing culturally-tailored public health messages. The HMI Framework—adapted from the National Institute on Minority Health and Health Disparities (NIMHD) [6] and grounded in social psychology, communication, and Human Centered Design—posits that creating effective public health messaging necessitates establishing a sustainable connection to BIPOC/MIMI, which requires addressing the underlying structural issues facing specific sub-segments. The HMI Framework guides the creation of public health messages for BIPOC/MIMI at the community and population levels, via the following sequential process:

1) *Identify the target population and sub-segments:* The cornerstone of HMI's approach is a nuanced and strategic approach to reaching BIPOC/MIMI. This necessitates a messaging approach that conceptualizes marginalized communities as heterogeneous rather than monolithic.
2) *Identify barriers and challenges:* HMI identifies barriers and challenges for the overall target population as well as the target sub-segment(s), prioritizing stakeholder and community outreach in order to accomplish this goal. This may include the deployment of a diverse range of participatory strategies—including stakeholder interviews, system mapping, and qualitative research—selected based on existing resources and project goals.
3) *Determine target outcomes:* Taking into account the population and sub-segment needs, experiences, and challenges, HMI devises messaging approaches to achieve Public Health Emergency Preparedness (PHEP) outcomes to facilitate the effectiveness of messaging on behavioral change [7].

**Figure 1:**
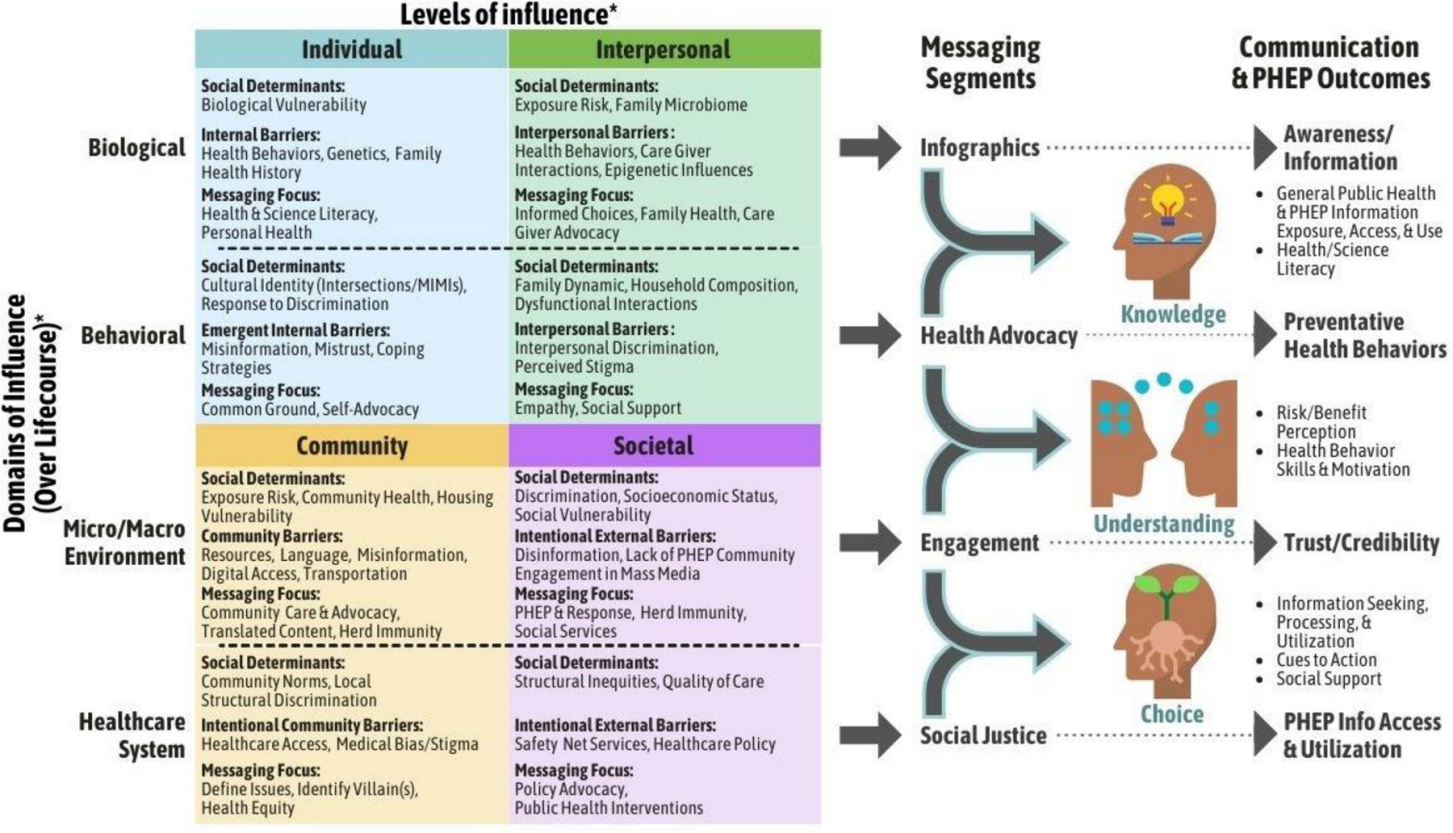
HMI Public Health Messaging Framework. This conceptual model—adapted from NIMHD [6]—reflects social, biological, and behavioral factors that inform the development of communications that promote health among BIPOC/MIMI. Delineated across levels of influence (Individual, Interpersonal, Community, Societal) and domains of influence (Biological, Behavioral, Micro/Macro Environment, Healthcare System) are relevant social determinants and barriers along with the messaging focus for each pairing to provide rationale for the associated messaging segments (Infographics, Health Advocacy, Engagement, Social Justice). The founding philosophy of HMI highlights the focus on pathways from perceptions to behavior to outcomes (Awareness/Information, Preventative Health Behaviors, Trust/Credibility, PHEP Info Access & Utilization) in promoting health literacy and self-advocacy as part of building behavioral skills and motivating health behavior change. This conceptualization is not intended to be exhaustive and may evolve over time.

HMI also deploys a range of strategies to maximize effectiveness across all steps.

1) *Community connection*: HMI engages with trusted messengers and community organizers at each step. HMI also signals allyship to the target communities and provides a platform to collaborate on messaging. This ensures that approaches are aligned with communities’ needs and promote activism within communities.
2) *Data-driven approaches:* HMI integrates data-driven and algorithmic approaches to complement community-oriented efforts. This includes the use of natural processing research protocols to analyze digital chatter, understand messages that are most resonant with population sub-segments, and iteratively revise the messaging strategy.
3) *Wide-scale dissemination strategies:* HMI utilizes multiple virtual and technological platforms, including podcasts, blogging, targeted social media engagement, satellite radio programming, webinars, and video infographics (examples provided in Table 1). In these efforts, partnerships with media platforms and civic organizations have promoted viewership, engagement, and increased access to relevant audiences.

**Table 1:** HMI Infographics Samples

Messaging Focus	Description	Example
**Health & Science Literacy**	Communicating information pertaining to the COVID-19 virus and vaccine, public health and safety, and government regulations and guidelines in an accessible and easy-to-understand manner.	“COVID-19 Vaccines & Herd Immunity.” (Posted on 4/29/2021; https://www.instagram.com/p/COQe1W2Liki/?utm_source=ig_web_copy_link)
**Personal Health**	Emphasizing conscious decisions to improve physical, mental, and spiritual self-care, including safe hygiene and health practices.	“There are 3 holes in yo face. Cover ALL of them. This ain't it fam.” (Posted on 4/3/2021; https://www.instagram.com/p/CNOESHDrgXc/?utm_source=ig_web_copy_link)
**Common Ground**	Centering the issues of COVID-19 as shared challenges faced by all communities, as well as the importance of addressing possible inequities together.	“Vaccine Diplomacy. A look at the pandemic crisis in India and the importance of ensuring global vaccine equity.” (Posted on 6/7/2021; https://youtu.be/VU9yFmCOAms)
**Self-Advocacy**	Promoting individuals and communities to practice and fight for equitable and safe standards within their respective settings.	“Making sure your school is doing the MOST.” (Posted on 7/27/2021; https://www.instagram.com/p/CR2SnnNqHFc/?utm_source=ig_web_copy_link)
**Community Care & Advocacy**	Promoting the health and advocacy of underserved communities and highlighting the individual's potential role and impact they may have on their community, and vice-versa.	“COVID is not the only plague of our era…hate is just as contagious as COVID. Stop spreading it!” (Posted on 4/11/2021; https://www.instagram.com/p/CNiWFV4LkYZ/?utm_source=ig_web_copy_link)
**Translated Content**	Translating English content into other languages to improve accessibility for non-English speaking communities.	“La ivermectina no esta aprobada para COVID (Ivermectin is not approved for COVID).” (Posted on 9/4/2021; https://www.instagram.com/p/CTLXuA3oOqH/)
**Herd Immunity**	Educating individuals on the science of herd immunity and the importance of vaccinations to protect oneself and others in the community, especially immunocompromised individuals and those without access to the vaccine yet.	“If it were the zombie virus…you would eat your whole family! Stop giving the virus new hosts!” (Posted on 7/5/2021; https://www.instagram.com/p/CQ8_7M_KYXU/)
**Define Issues**	Defining both the explicit and implicit social, political, and public health issues that contribute to inequities with regard to the COVID-19 pandemic and vaccination efforts.	“Equitable care starts with CARE providers…you AREN'T a good doctor if you can't diagnose your own biases.” (Posted on 4/28/2021; https://www.instagram.com/p/COOHtCaqFM2/)
**Informed Choices**	Encouraging individuals to make scientifically informed and healthy decisions that are specific to themselves as well as their families and communities.	“The CDC updated mask & social distancing guidelines…they say…if you're fully vaccinated…you can take your mask off indoors & outdoors. But do you really want to?” (Posted on 7/2/2021; https://www.instagram.com/p/CQ6Nb92qfZo/)
**Empathy & Social Support**	Offering supportive resources (including emotional and mental comfort) in light of sociopolitical and public health challenges and disparities.	“Pump Up Those Antibodies! COVID-19 vaccines are safe for pregnant & lactating mothers...” (Posted on 8/29/2021; https://www.instagram.com/p/CTLLqGgKCiY/)
**PHEP & Response**	Broadcasting of Public Health Emergency Preparedness communications and response to ensure individuals are adequately prepared throughout the pandemic.	“CAUTION. There are still settings where you should mask regardless. There are still mutant variants…everywhere, which could cause these guidelines to change.” (Posted on 5/19/2021; https://www.instagram.com/p/CPEQsr6lFkF/)
**Identify Villain(s)**	Identifying the causes or antagonists that perpetuate dis/misinformation, inequities, and other barriers for BIPOC/MIMI.	“We get it. You don't want to get vaccinated. Feels a lot like Tuskegee… Tuskegee is just one of America's many crimes against humanity involving unethical human experimentation & patient cruelty. It's hard to separate the instinctive apprehension these acts provoke in all of us from the reality of how science is conducted in the modern era. Contemporary medical bias, racism, and lingering apartheid have corrupted our trust in health professionals…” (Posted on 7/27/2021; https://www.instagram.com/p/CR1Vhuolckr/)

Examples of segmented messaging relating to the PHEP and other health behavior outcomes. Posting dates refer to the organizational Instagram account (https://www.instagram.com/hood_medicine/).
4) *Iterative message refinement:* HMI messaging is continually refined via ongoing collaborations and dialogues with community members and leaders as well as epidemiological findings.

The pandemic was a stark reminder of the persistent structural disparities endured by BIPOC/MIMI. Targeted mal/dis/misinformation by various anti-vaccine organizations and other actors further accelerated inequities [8]. Ultimately, both non-governmental and governmental organizations (e.g., the Rockefeller Foundation) stepped in to try to address these inequities and the Biden Administration formed a Health Equity Task Force—comprised of senior leadership in health equity [9,10]—in August 2021 to address COVID-19 inequities. With this increased momentum, we must prioritize approaches that are both community-oriented and data-driven. Adapting and scaling the HMI model holds potential for combating health disparities for BIPOC/MIMI during and post-pandemic.

## Declaration of Competing Interest

The authors have no declarations to make.
